# Supplementary material for: A set of multi-entry identification keys to African frugivorous flies (Diptera, Tephritidae)
Source: Zookeys. 2014 Jul 24;(428):97–108. doi: 10.3897/zookeys.428.7366 (PMC4143993; doi:10.3897/zookeys.428.7366)
Supplement: Supplementary material 6 — Key to Ceratitis [file zookeys-428-097-s006.zip › SF6_ZooKeys_key to Ceratitis/key/SF6_ZooKeys_key to Ceratitis/Media/Html/Ceratitis munroanum.htm]

***Ceratitis*** **(*Acropteromma*)
*munroanum* (Bezzi)**

*Acropteromma* *munroanum*
Bezzi, 1926: 281.

Body length: 6.23 (5.80-6.80) mm; wing length: 6.80 (6.30-7.30)
mm.

Male

Head.
Antenna yellow-orange. First flagellomere twice as long as pedicel. Arista
almost completely bare, with a few very short hairs at basal part dorsally.
Frons convex, in lateral aspect not projecting at antennal implant;
yellow-white, sometimes darker yellow-orange; with short scattered hairs
about same colour as frons. Two weakly developed pale
frontal bristles. Two well-developed but
relatively short black orbital bristles. Face yellow-white. Genal bristle long
and pale; genal setulae pale, well developed.

Thorax.
Postpronotum white, around bristle slightly yellow, but without distinct spot.
Scutum, ground colour orange with greyish tinge; with streaks and darker
markings but no distinct spots or clearly defined stripes. Scapular setae dark.
Scutellum yellowish white, basally with yellow spots; apically with three
separate black spots, reaching to basal third. Anepisternum yellow-white, lower
half darker yellow; one black anepisternal bristle, otherwise pale pilosity.
Subscutellum pale.

Legs. Yellow to yellow-orange. Fore femur posteriorly with well-developed orange bush of hairs; ventral bristles orange or darkish.
Otherwise with mainly pale pilosity, rarely partially dark.

Wing.
No banding or basal spots. Apical tip pointed and with distinct black spot.
Crossvein r-m at middle of discal cell. Discal cell broad. Crossvein dm-cu posteriorly more inwards than anteriorly.

Abdomen. Yellow to orange. Tergites 2 and 4 greyish, except
anterior margin.

Female

As male, except for the following characters. First flagellomere
longer, about three times as long as pedicel. Arista with slightly longer hairs.
Frontal bristles black and well-developed. Genal seta and setulae pale or
darkish. Fore
femur without distinct orange bush, ventral spines black. Wing, apically not
pointed and spot weakly developed. Ovipositor shorter
than abdominal tergites.�

(Redescription
after De Meyer & Copeland, 2001)
